# The distribution of cardiac diagnostic testing for acute coronary syndrome in the Brazilian healthcare system: A national geospatial evaluation of health access

**DOI:** 10.1371/journal.pone.0210502

**Published:** 2019-01-10

**Authors:** Julian T. Hertz, Tommy Fu, Joao Ricardo Vissoci, Thiago Augusto Hernandes Rocha, Elias Carvalho, Brendan Flanagan, Luciano de Andrade, Alex T. Limkakeng, Catherine A. Staton

**Affiliations:** 1 Department of Surgery, Division of Emergency Medicine, Duke University Medical Center, Durham, North Carolina, United States of America; 2 Duke Global Health Institute, Duke University, Durham, North Carolina, United States of America; 3 Department of Medicine, Centro Universitario Inga, Maringa, Brazil; 4 Panamerican Health Organization, Brasilia, Brazil; 5 Department of Computer Science, Pontifical University Catholic of Parana, Curitiba, Brazil; 6 Data Processing Department, State University of Maringa, Maringa, Brazil; 7 Department of Medicine, State University of Maringa, Maringa, Brazil; Western University, CANADA

## Abstract

**Background:**

Little is known about the utilization of cardiac diagnostic testing in Brazil and how such testing is related with local rates of acute coronary syndrome (ACS)-related mortality.

**Methods and results:**

Using data from DATASUS, the public national healthcare database, absolute counts of diagnostic tests performed were calculated for each of the 5570 municipalities and mapped. Spatial error regression and geographic weighted regression models were used to describe the geographic variation in the association between ACS mortality, income, and access to diagnostic testing.

From 2008 to 2014, a total of 4,653,884 cardiac diagnostic procedures were performed in Brazil, at a total cost of $271 million USD. The overall ACS mortality rate during this time period was 133.8 deaths per 100,000 inhabitants aged 20 to 79. The most commonly utilized test was the stress ECG (3,015,993), followed by catheterization (862,627), scintigraphy (669,969) and stress echocardiography (105,295). The majority of these procedures were conducted in large urban centers in more economically developed regions of the country. Increased access to testing and increased income were not uniformly associated with decreased ACS mortality, and tremendous geographic heterogeneity was observed in the relationship between these variables.

**Conclusions:**

The majority of testing for ACS in Brazil is conducted at referral centers in developed urban settings. Stress ECG is the dominant testing modality in use. Increased access to diagnostic testing was not consistently associated with decreased ACS mortality across the country.

## Introduction

Acute coronary syndrome (ACS) and other forms of cardiovascular disease remain one of the leading causes of death and morbidity worldwide, currently accounting for one of every three deaths [1]. One of the challenges of diagnosing ACS is the tremendous range of diagnostic options. Symptoms frequently overlap with more benign conditions and health institutions have different degrees of technological sophistication. Diagnostic evaluation is typically a combination of electrocardiogram (ECG), laboratory testing (troponin, CK-MB), cardiac catheterization, and functional testing, which is commonly referred to as the stress test [2]. Studies have demonstrated that in patients with cardiovascular risk factors, a stress test can accurately identify those at risk of cardiac events in the near future [3–5]. Despite the widespread use of stress tests, there has been a resurgent debate over the proper role of stress testing in the evaluation of patients with possible ACS: some have argued that stress testing provides valuable prognostic information and risk-stratification in appropriate patients while others have argued that stress testing can produce both false positive and false negative results and does not reduce mortality [6, 7].

Globally, the incidence of ACS is rising with better treatment of infectious diseases and development-related lifestyle changes that predispose to heart disease, such as obesity, hypertension, and diabetes [1]. Brazil is a country that is rapidly developing and provides an intriguing case study in cardiovascular resource utilization. Rapid economic development in Brazil has introduced changes in diet and exercise patterns that have made cardiovascular disease increasingly prevalent, even in previously low risk populations such as the young [8, 9]. Recent studies have shown that the rate of obesity in Brazil has more than tripled in the past 40 years [10, 11]. In 2011, cardiovascular disease accounted for 31% of all deaths in Brazil (7% from ACS alone) and 19% of total spending on hospitalizations (580 million USD) [8, 12]. As cardiovascular disease becomes more prevalent, various forms of stress testing are becoming increasingly available across the country, including cardiac catheterization, stress ECG, stress echocardiography, and stress scintigraphy [5, 13]. The clinical purpose of these tests is to detect decreased coronary blood flow during stress, either by ischemic ECG changes (stress ECG), focal wall motion abnormalities (stress echocardiography), decreased perfusion (stress scintigraphy), or direct visualization of the coronary arteries via angiography (cardiac catheterization). Each stress test modality has its relative merits, but uniform guidelines about the proper use of each test are lacking in Brazil, and so choice of stress test may depend on local resource availability, patient comorbidities, and physician preference. In one small study examining the utilization of exercise stress tests in Northern Brazil, the vast majority of stress tests were found to be inappropriate and fueled by higher availability within private healthcare systems rather than pre-test probability or existing risk factors [14]. On a national level, however, little is known about how patients in Brazil are risk stratified for ACS, how these methods are changing, and whether these methods are effective. Specifically, there are no national-level studies on the utilization of and accessibility to various stress tests across different locales and sociodemographic groups.

Brazil’s unified national healthcare system has developed several national health information systems containing comprehensive nationwide health-related data, which allows for the unique opportunity to perform large ecological studies about health systems challenges and outcomes. These national databases contain detailed registries of procedures, diagnoses, costs, deaths, and patient demographics for the entire country [15]. The availability of such comprehensive data in a setting with tremendous income disparities makes Brazil a perfect setting for an analysis of the interaction between healthcare access, socioeconomic inequality, and outcomes. To our knowledge, no previous study has been conducted in a low or middle income country to analyze the association between the geospatial distribution of access to stress testing, income, and ACS mortality. The purpose of this study is to characterize the geographic distribution of stress testing in Brazil, and describe the association of access to diagnostic testing with ACS mortality. The results are anticipated to inform the role of stress testing in other developing nations as well.

## Methods

### Study design

This observational, cross-sectional, and ecological study utilized secondary data from 5570 Brazilian municipalities with spatial analysis techniques to investigate geographic association of ACS testing and mortality from 2008 to 2014. This time period was chosen due to changes in data collection systems that led to improved data quality beginning in 2008 [16]; 2014 is the last year for which mortality data is available. This protocol was submitted to the Duke University Institutional Review Board and was exempted from full board review due to the de-identified nature of the data. Exploratory spatial data analysis was used to assess the relationship between cardiac diagnostic test utilization and ACS mortality rate across Brazil using data from DATASUS, the public national healthcare database.

### Study population and location

Brazil is the largest country in South America, occupying an area of 8,516,000 square kilometers with a total population of 190,732,694 persons, according to the 2010 Brazilian Census [17]. Brazil’s emerging economy has undergone recent rapid expansion with a gross domestic product (GDP) of 1,803 billion USD in 2015, making it the ninth largest economy in the world [18]. This rapid economic development, however, has occurred very unevenly across different regions of Brazil, leading to tremendous income inequality across the country [19].

### Sociodemographic and socioeconomic data

**Fig 1:**
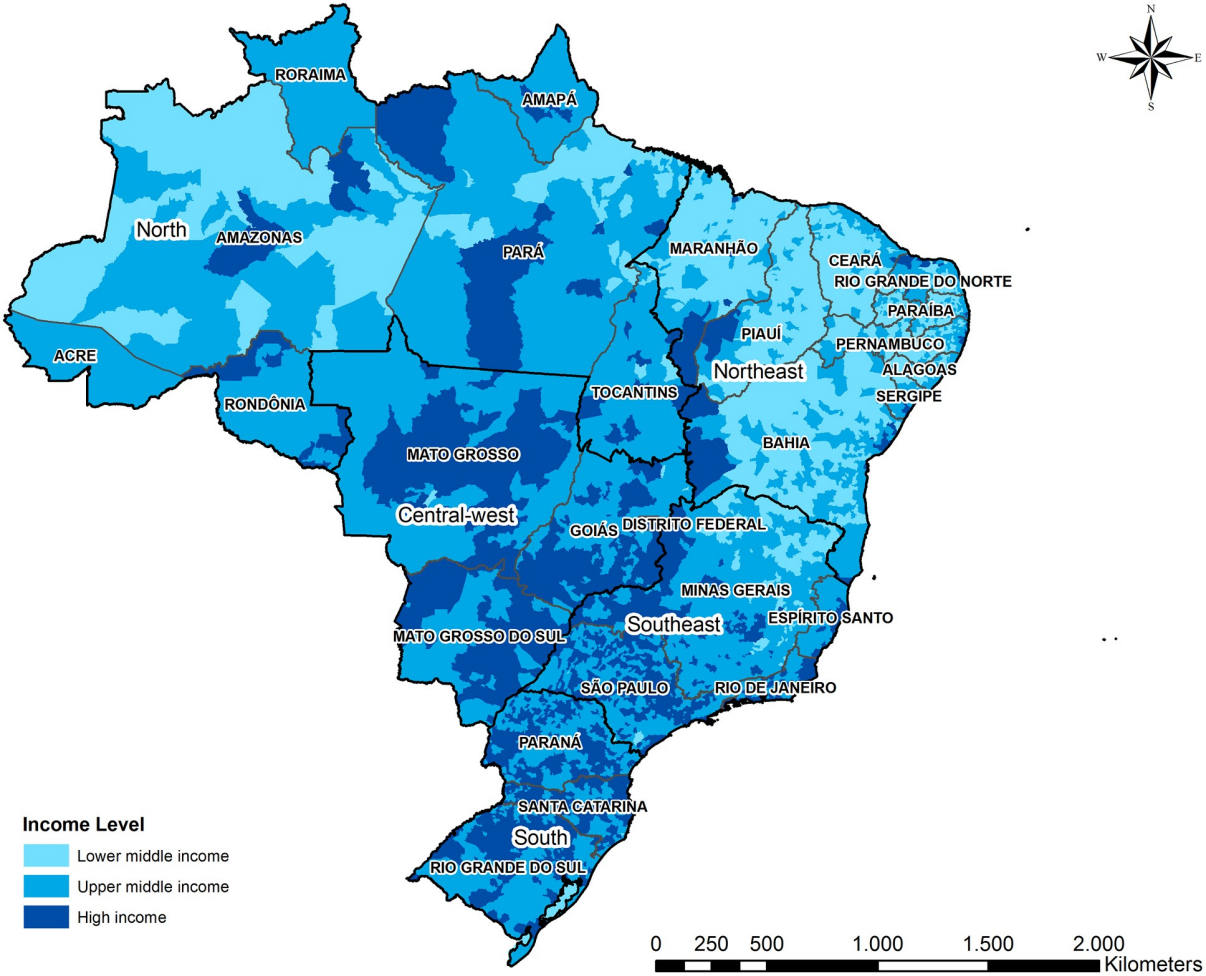
Brazilian regions and municipality income levels.

Socioeconomic data, specifically the gross domestic product per capita, at the municipality level were extracted from Brazilian Institute of Geography and Statistic (IBGE) [17] and the World Bank [19]. Each Brazilian municipality was classified to income level following the protocols used by the World Bank income classification [20]. The World Bank’s atlas index, together with data regarding the Brazilian GDP, was used to stratify all municipalities of the country as high income, upper middle income, or lower middle income. No Brazilian municipality met the World Bank definition for low income. Fig 1 depicts the income levels of all municipalities in Brazil as defined by the World Bank’s atlas index.

### ACS diagnostic procedures and mortality data

Testing rates per population of cardiac catheterization, stress electrocardiography (ECG), scintigraphy, and stress echocardiography were compiled by municipality from the Ambulatory Information System database (SIA) [21] (Table 1). The SIA database is dedicated to monitoring the number and cost of procedures performed in Brazilian public hospitals. Data regarding ACS-related mortality rates by municipality was obtained from the Mortality Information System database (SIM) [22], including all data tagged with the ICD 10 codes related to ACS (I20, I21, I22, I23, I24 and I25). Mortality cases were ascribed to the home municipality where the decedent resided, not the municipality in which the death occurred. For the calculation of adjusted mortality rate, only the municipality population between ages 20 and 79 years was included, in accordance with American Heart Association recommendations [23].

**Table 1:** Data sources for analysis.

Source	Variables	Date Range	Data entries	Scope
DATASUS—Ambulatory Care System (SIA)	Healthcare procedures performed Associated ICD codes Age of patient Location offering the procedure Costs of procedure Hospital responsible	2008–2014	4,653,884 procedures	Catheterization Scintigraphy Stress Echocardiography Stress ECG
DATASUS—Mortality Information System (SIM)	Cause of death coded by ICD groups: I20, I21, I22, I23, I24, and I25 Municipality of residence of patients with ACS death Mortality rate by municipality.	2008–2014	714,670 deaths	All deaths between 2008 and 2014
CNES -National Registration of Health Establishments	Geolocation Procedures offered	2008–2014	1672 hospitals	Hospitals performing ACS diagnostic procedures between 2008 and 2014
World Bank	Gross national income Atlas index Gross national income per capita Income level classification	2010–2013	5570 municipalities	
IBGE—Brazilian Institute of Geography and Statistics	Population by municipality—between 20 and 79 years of age Gross Domestic Product (GDP) GDP per capita	2008–2014	5570 municipalities	

### Health infrastructure data

**Fig 2:**
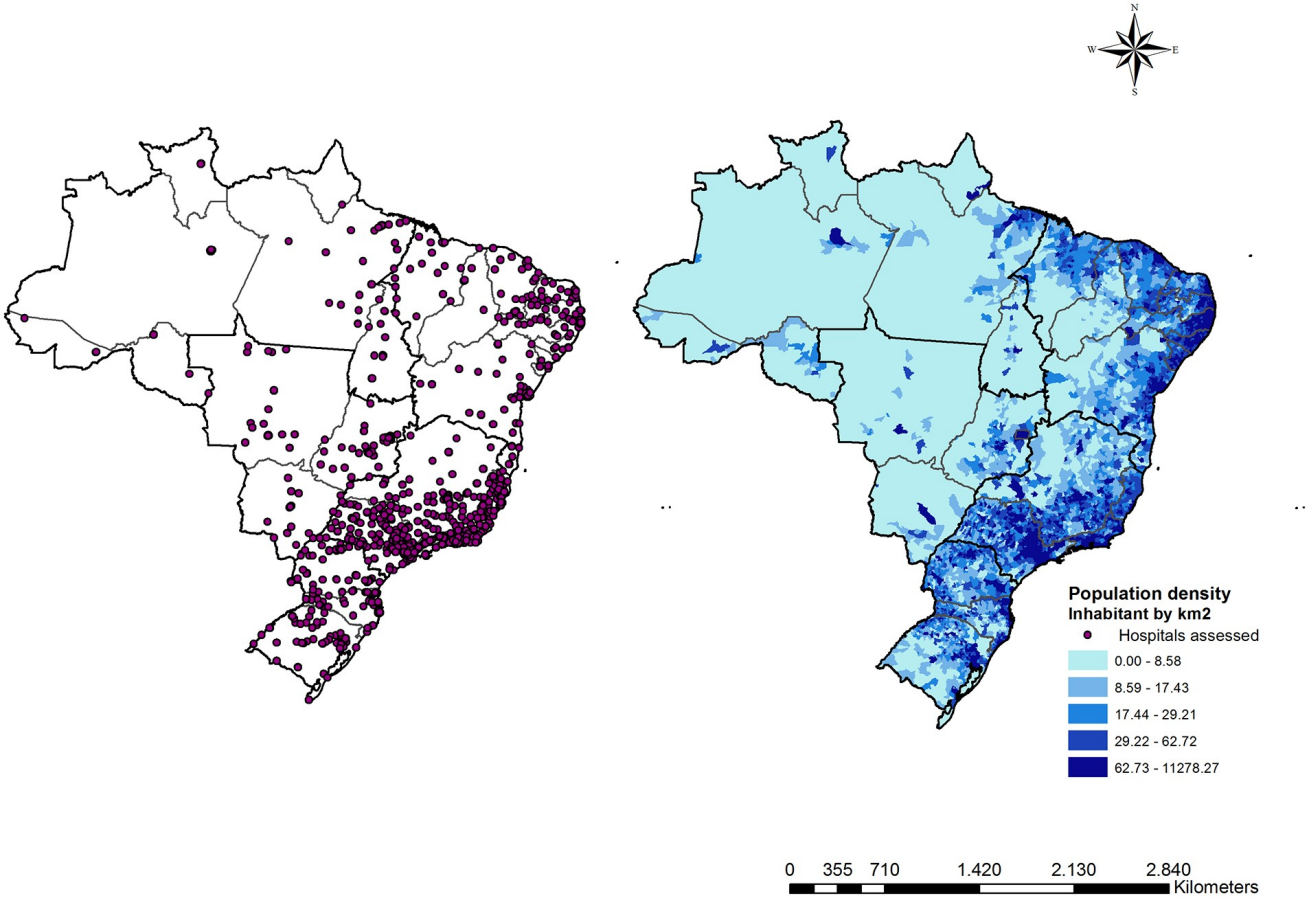
The distribution of hospitals performing cardiac diagnostic testing and the distribution of population density in Brazil.

Additional data about public hospitals performing cardiac diagnostic procedures (stress ECG, stress echocardiography, scintigraphy, and catheterization) were obtained. Geolocation and infrastructure data were obtained from Cadastro Nacional de Estabelecimentos de Saúde / National Register of Health Facilities [24]. Fig 2 presents the distribution of hospitals performing cardiac diagnostic testing in Brazil as well as the distribution of population density across the country.

### Data analysis

All socioeconomic and health indicators were normalized to the Brazilian population. Conversion from Brazilian reals (BRL) to US dollars (USD) was performed using 2017 values. ACS-related mortality rates, diagnostic procedure rates, and cost data were summarized using descriptive statistics. The population between 20 and 79 years of age was selected to adjust the mortality and procedure rates by 100,000 persons, as per American College of Cardiology and American Heart Association guidelines [23]. All analyses were performed at a significance threshold of 0.05.

### Geographical accessibility to ACS diagnostic procedures: Accessibility index

To evaluate geographical availability of ACS diagnostic procedures, the two-step floating catchment area (2SFCA) method was used [25]. The 2SFCA technique creates an index for availability and proximity of ACS diagnostic testing weighted by population in two concurrent steps. In the first step, a buffer with a 120 kilometer (km) radius surrounding each public hospital was created to collect the population potentially covered. The 120 km range limit was used to estimate a travel distance of two hours to nearest cardiac testing center, the maximum distance recommended by the American Heart Association and European Society of Cardiology [26]. A capability index for each hospital was calculated by dividing the average number of cardiac diagnostic tests performed at each center by the population within the 120 km buffer surrounding the hospital for each year. The equation is:

$$R_{j}=\frac{S_{j}}{\sum_{k\in \{d_{kj}\leq d_{0}\}}\frac{D_{k}}{1000}}$$

where d _kj_ is the distance between k and j, D _k_ is the demand at location k that falls within the catchment, and S _j_ is the capacity of supply of cardiac diagnostic testing performed at location j [27].

In the second step, the capability indices of all hospitals within 120 km from each municipality’s centroid were combined. For each demand location i, we search all supply locations j that are within the threshold distance d_0_ from location i and sum up the supply-to-demand ratios Rj at those locations to obtain the full accessibility A_i_^F^ at demand location i. The final equation of accessibility index is [27]:

$$A_{i}^{F}=\sum_{j\in \{d_{ij}\leq d_{0}\}}R_{j}=\sum_{j\in \{d_{ij}\leq d_{0}\}}\left(\frac{S_{j}}{\sum_{k\in \{d_{kj}\leq d_{0}\}}\frac{D_{k}}{1000}}\right)$$

This produced an *accessibility index* of cardiac diagnostic procedures for *each municipality*. A higher accessibility index indicates more access to cardiac diagnostic testing for a given municipality. The 2SFCA method was performed for each of the four procedures considered (cardiac catheterization, stress echocardiography, stress ECG, and nuclear scintigraphy) using the average rate of procedures between 2008 and 2014. Choropleth maps were generated using software ArcGIS 10.5 to visualize the accessibility index to the ACS diagnostic procedures.

### Association between mortality and accessibility

Geospatial based regression models were used to model the effect of income and accessibility indices of each diagnostic modality on ACS mortality. These models use multivariate regression to evaluate associations between predictors (income, accessibility indices of diagnostic modalities) and ACS mortality rate taking into account any spatial dependency. Spatial dependency is observed when one variable behavior (e.g. ACS mortality) in one given geographical space (municipality) is dependent on the behavior of that variable in the neighboring geographic spaces. In other words, spatial dependency occurs when the likelihood of a municipality having a high ACS mortality rate coincides with high ACS mortality rates of its neighboring municipalities. The Jarque-Bera method was used to test the multivariate normality of the residuals of the models, while the Breush-Pagan and Koenker-Basset tests were used to evaluate heteroscedasticity. Finally, Moran’s I and the Lagrange multiplier test were used to evaluate the spatial dependency of the models.

Initially, we analyzed the data with an Ordinary Least Squares model (OLS) without taking into account the spatial dependency. Given that the data showed high spatial autocorrelation of the residuals, two geospatial regression techniques were used. The first technique was the spatial error regression model which produces a regression model where the spatially correlated errors are controlled for and included in the model as an indicator. This analysis performs a global analysis of the entirety of the Brazilian geographical area, adjusting for the geospatial dependency.

However, since the spatial error model still showed high heteroscedasticity, measured by the Koenker-Basset test, we used the second geospatial regression technique. The Geographically Weighted Regression (GWR) model, which controls for non-stationarity by conducting multiple separate post hoc regressions for sub-areas within the global geographical area (Brazil). The fixed distance bandwidth considered for the GWR analysis was 363 kilometers. Each separate regression model is calculated aggregating the municipalities that are clustered together based on their outcome behavior (ACS mortality). This method yields an estimation for the association of ACS mortality rate and the predictors for each municipality. The performance of the GWR model was evaluated based on the adjusted R2 indicators, Akaike’s information criterion parameters (AICc) and Moran’s I of the residuals. Sensitivity analyses were conducted by comparing the model to a standard Ordinary Least Squares (OLS) model. The spatial self-correlation and OLS model were processed using GeoDa software 1·10·0·8 (Spatial Analysis Laboratory, Urbana, IL), and the GWR model by GWR 4·0.22. Choropleth maps were created using QGIS 2·14 software.

## Results

From 2008 to 2014, there were 714,670 ACS-related deaths in Brazil with an overall mortality rate of 133.8 deaths per 100,000 inhabitants between 20 and 79 years of age. Most of the deaths occurred in the South, Northeast and Southeast regions (Table 2). The North and Center-West regions accounted for a lower proportion of ACS-related deaths. The ACS mortality rate was highest in the South (158.6 deaths/100,000 inhabitants between 20 and 79 years).

**Table 2:** Characteristics of stress testing in Brazil and regional distribution.

		Brazilian regions					
Procedures		Center-west	Northeast	North	Southeast	South	Brazil
**Catheterization**	**N (%)**	55,633 (6.4%)	152,441 (17.7%)	26,621 (3.1%)	458,199 (53.1%)	169,733 (19.7%)	862,627 (100%)
	**N/100k inhabitants (SD)**	205.7 (57.7)	340.7 (69.6)	201.5 (35.3)	387.5 (87.1)	545.5 (114.5)	399.4 (87)
	**Cost in USD**	10,183,369	27,973,972	4,870,375	83,828,598	31,033,846	157,890,161
**Stress Echo**	**N (%)**	13,683 (13.0%)	18,153 (17.2%)	13,474 (12.8%)	44,708 (42.5%)	15,277 (14.5%)	105,295 (100%)
	**N/100k inhabitants (SD)**	180.9 (120.5)	279.3 (183.7)	341.7 (92.9)	123 (89.8)	72.8 (58.1)	153.1 (103.6)
	**Cost in USD**	883,941	882,888	660,668	2,161,854	741,813	5,332,164
**Scintigraphy**	**N (%)**	27,221 (4.1%)	146,803 (21.9%)	2,3790 (3.6%)	378,403 (56.5%)	93,752 (14.0%)	669,969 (100%)
	**N/100k inhabitants (SD)**	168.5 (86.3)	271.7 (78.5)	181 (99.7)	258 (65.1)	194.3 (79.8)	237.3 (73.4)
	**Cost in USD**	3,259,379	17,625,845	2,848,253	44,953,883	11,171,408	79,858,768
**Stress ECG**	**N (%)**	212,782 (7.1%)	551,655 (18.3%)	128,935 (4.3%)	1,706,256 (56.6%)	416,365 (13.8%)	3,015,993 (100%)
	**N/100k inhabitants (SD)**	648.3 (412.7)	893.3 (555.3)	474.9 (283.8)	905.9 (426.4)	660.4 (294.2)	815.7 (419)
	**Cost in USD**	2,860,012	5,183,218	1,151,205	15,239,763	3,723,223	28,157,422
**ACS**	**Total number of deaths**	79,092	328,164	55,286	618,667	209,267	1,290,476
	**Mortality rate per 100k**	127.6	139.4	84.5	125.0	158.6	133.8

During this time period, a total of 4,653,884 cardiac diagnostic procedures were performed in Brazil, at a total cost of $271 million USD (Table 2). These procedures were conducted in 1,672 healthcare facilities. The most commonly utilized test was the stress ECG (3,015,993), followed by catheterization (862,627), scintigraphy (669,969) and stress echocardiography (105,295) (Table 2). The procedures rates, weighted by population, demonstrated geographic imbalances in testing distribution among Brazilian regions. The rates of ACS diagnostic testing performed by population were different depending on the procedure considered. Catheterization rates were highest in the higher-income Southeast and South regions, whereas stress echocardiography rates were highest in the lower-income North and Northeast regions. Rates of scintigraphy and stress ECG testing were highest in the Northeast and Southeast regions.

**Fig 3:**
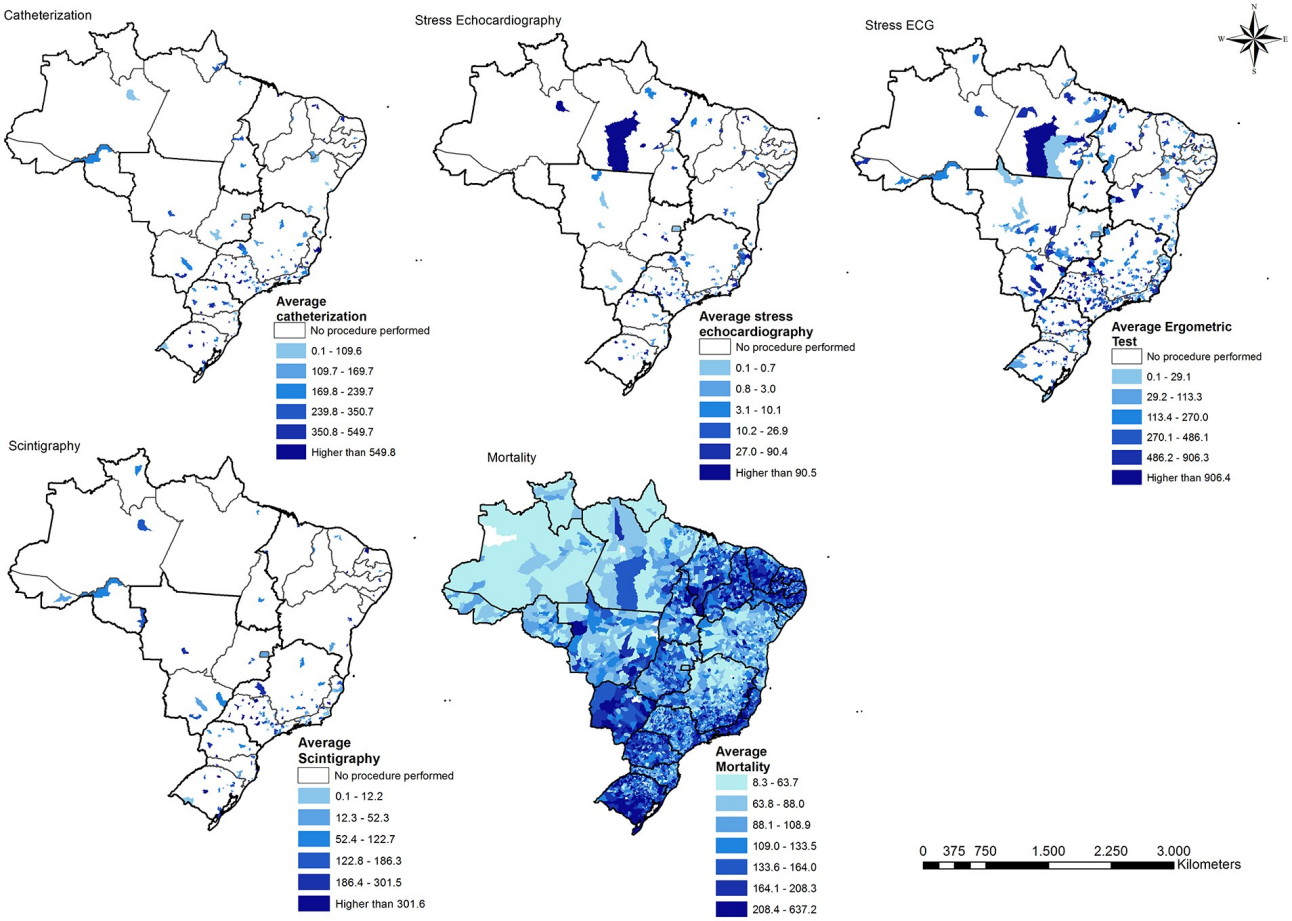
Distribution of cardiac diagnostic testing rates and ACS mortality rates in Brazil, 2008–2014 (per 100,000 persons).

The spatial distribution of the four ACS diagnostic modalities and ACS-related mortality rates observed from 2008 until 2014 are presented in Fig 3. Catheterization and scintigraphy, in particular, were mainly performed in municipalities of the South and Southeast regions. Stress ECG testing, the most commonly performed diagnostic procedure, was more widely distributed across the country. White spaces in the figure represent areas where procedures were not performed, suggesting a lack of access. The distribution of ACS-related mortality rates demonstrates a trend of particularly high mortality rates in the South, southern and coastal Southeast, southern Mid-west and Northeast regions.

**Fig 4:**
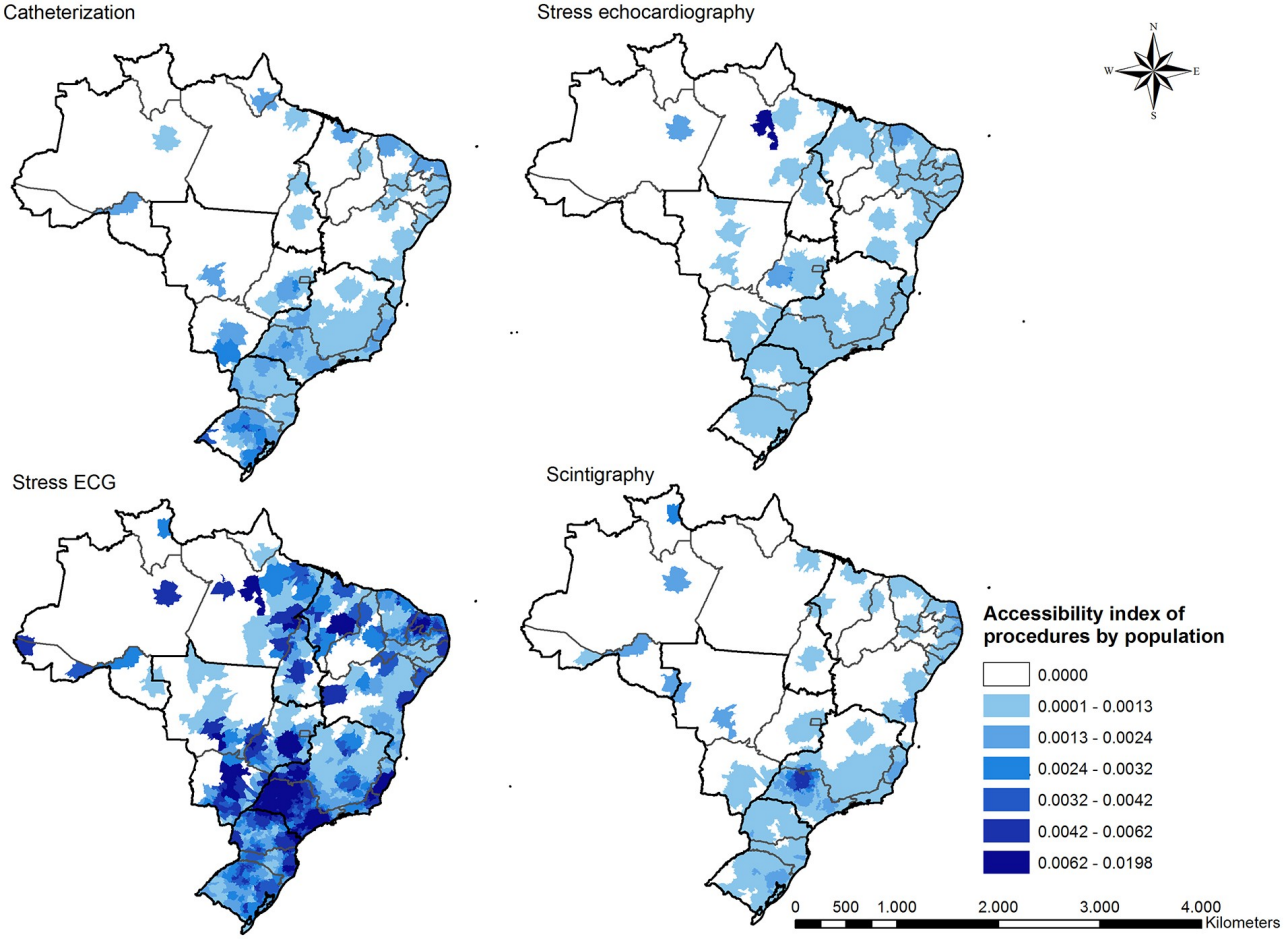
Geographic variation in access to cardiac diagnostic testing in Brazil.

The accessibility indices for cardiac diagnostic procedures were higher in the more economically developed regions of the South and Southeast regions (Fig 4). Stress ECG had the highest overall accessibility index but even access to this test was still distributed very unevenly. It is also noteworthy that large portions of Brazil (Central-west, North, and rural Northeast) do not have access to three of the four diagnostic procedures analyzed, despite in some cases having relatively high rates of ACS mortality (Figs 3 and 4). Furthermore, even in regions with abundant access to stress testing (South, Southeast), testing centers tended to be clustered in large cities, leaving those outside large cities with poor access.

The association between ACS-related mortality, income, and accessibility of cardiac diagnostic modalities tested through the unadjusted OLS model showed high spatial dependency, with low adjusted R-squared and higher AIC. The unadjusted OLS model found that higher ACS-related mortality was associated with lower income, lower access to scintigraphy, higher access to catheterization and high access to stress ECG testing (Table 3). However, when controlling for the high spatial dependency in the spatial error model, higher ACS-related mortality was associated with increased access to catheterization and decreased access to scintigraphy. The association between income and ACS mortality was not significant in the spatial error model. The spatial error model had a markedly higher adjusted R-squared value, indicating better fit (Table 3).

**Table 3:** Regression coefficients of income and cardiac diagnostic testing accessibility as predictors of ACS mortality in unadjusted ordinary least square model, model adjusted for spatial error, and geographic weighted regression model.

	Ordinary Least Square			Spatial Error			Geographic Weighted Regression	
Variables	Est.	SE	*p*	Est.	SE	*p*	Est. Mean	Est.SD
Income	-4.49	1.47	0.002*	2.05	1.67	0.219	4.93	56.19
Catheterization accessibility	18627.70	1620.08	<0.001*	8937.12	2585.24	0.001*	-24639.31	555297.72
Stress Echocardiographyaccessibility	-755.51	2377.47	0.751	556.02	3091.71	0.857	1448563.77	6735792.94
Scintigraphy accessibility	-13457.10	1786.88	<0.001*	-8890.07	2999.31	0.003*	-2059.62	932050.69
Stress ECG accessibility	1783.68	396.38	<0.001*	769.99	651.23	0.237	14741.88	108212.70
AIC	63348.2			61896.1			3410.32	
Jarque-Bera Statistic	2348.23		<0.001*					
Adj. R^2^	0.0300			0.2994			0.2851	
Koenker’s Statistic	34.14		<0.001*					
Breusch-Pagan Statistic	74.33		<0.001*	82.38		<0.001*		
Lag coefficient (Rho)				0.5963	0.0146	<0.001*		

*p < 0.05

**Fig 5:**
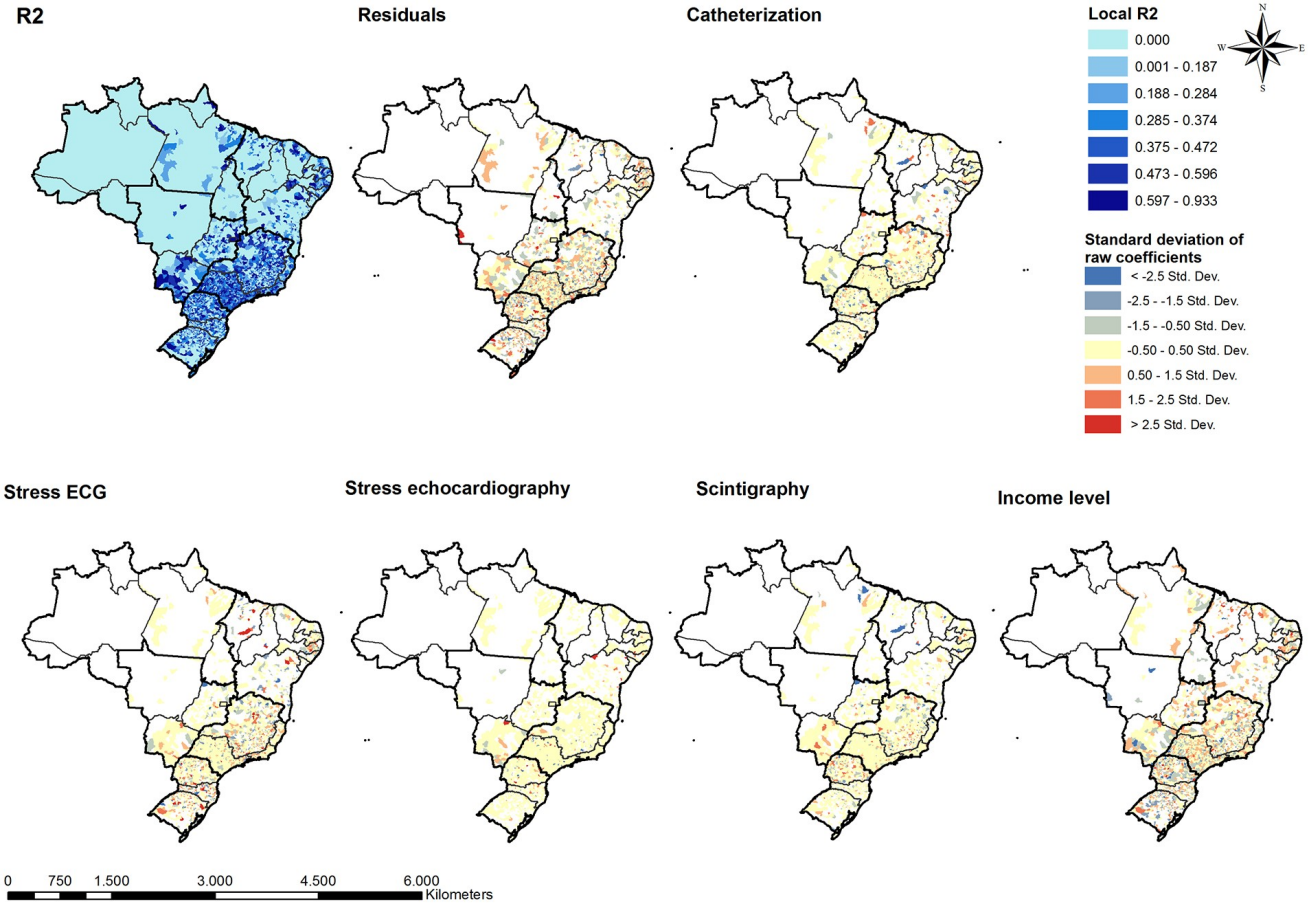
Coefficients of geographic weighted regression of income and accessibility indices of cardiac diagnostic testing as predictors of ACS mortality in Brazil, 2008–2014.

In the post hoc type analysis of the GWR model, higher ACS-related mortality rates were associated with higher income and increased access to stress echocardiography and stress ECG, as well as decreased access to catheterization and scintigraphy. However, the standard deviations of the estimated coefficients are large indicating an heterogeneous distribution of the locally estimated association. Fig 5 presents the distribution of the estimated coefficients from the GWR model. S1 Fig presents the t-value maps demonstrating locations where the GWR model found statistically significant association between predictor variables and ACS mortality. Inspection of these distributions highlights the geographically heterogenous relationship between ACS mortality, income, and access to testing. Increased access to diagnostic testing was generally associated with lower ACS mortality in the Northeast, whereas the association between ACS mortality and access to testing was more mixed in the South. The relationship between income and ACS mortality was also very heterogenous, without consistently positive or negative associations in any given region.

## Discussion

This is the first nation-wide analysis of the geospatial distribution of the utilization of various cardiac diagnostic modalities and its association with local ACS-related mortality. It is performed in Brazil, the fifth most populated nation in the world [28], with a unified nationalized health system. We did find that cardiac testing has generally been clustered in specific urban centers, that stress ECG is the dominant modality used across Brazil, and that there are several geographic areas with significant mismatch between ACS mortality and test accessibility. Overall, we found no geographically consistent association between cardiac testing, income, and ACS mortality. Given the size and depth of data available from of the Brazilian health system we believe these findings have identified several avenues for additional research and health system improvement for both Brazil and globally.

As expected with Brazil’s public health system’s centralization of testing facilities, accessibility to cardiac testing was largely limited to urban areas and was extremely variable across the country. Large segments of rural and underdeveloped areas of the country did not have access to either stress echocardiography, scintigraphy or cardiac catheterization, while there was abundant access to testing in urban centers such as Sao Paulo, Rio de Janeiro and other regions around large cities. The uneven distribution of test utilization observed in Brazil is not unique and has also been described in high income countries such as the United States [29]. Although there has been little study of the geographic distribution of cardiac testing in low and middle-income countries, testing capacity is also likely to be clustered in urban centers in these countries as well.

In our analysis, the relationship between ACS mortality and access to cardiac diagnostic testing was complex and not geographically uniform. Although stress testing is widely used in high income countries, recent evidence suggests that increased utilization of such testing is not associated with lower cardiovascular mortality in such settings [29–32]. To our knowledge, there has been no study of this association in low and middle income countries. Overall, in this study, no convincing association between access to diagnostic testing and decreased ACS mortality was found. Of all the diagnostic tests analyzed, only scintigraphy showed an overall association with decreased ACS mortality, but this association was not consistent across the country. Increasing access to the other diagnostic tests had either no overall association with ACS mortality or (in the case of catheterization) an association with higher ACS mortality, even when controlling for income. The fact that increased access to diagnostic testing did not have a geographically consistent association with decreased ACS mortality may suggest that even in upper middle income countries like Brazil where access to such advanced testing remains limited, increasing utilization of such testing may not lead to a mortality benefit. These findings are supported by studies in high income countries which have not found a mortality benefit from increased use of cardiac stress testing [29–32].

Although ACS mortality rates also tended to be higher in more urban areas in Brazil, access to testing and local ACS mortality rates were often not well matched. For example, large segments of the South, Southeast, Center West and Northeast regions of the country were characterized by relatively high rates of ACS-related mortality but very limited access to cardiac diagnostic testing. These underserved areas warrant particular attention from public health officials when planning to expand cardiac diagnostic testing. Expansion of the existing network of hospitals with advanced cardiac capabilities must be considered to ensure that all Brazilians can have prompt access to potentially life-saving procedures (particularly catheterization) in the event of cardiac emergencies. Alternatively, we must also consider that potentially prolonged wait times for diagnostic testing in the public system, inappropriate use of stress testing, differences in test sensitivity and specificity, and variable adherence to diagnostic algorithms could also potentially explain the observed absence of association between access to testing and ACS mortality. As such, if expansion were to occur, additional research is needed to determine the benefits of stress testing on cardiovascular mortality, or whether targeted strategies and locally-developed appropriate use guidelines might result in a reduction in morbidity and mortality in these countries. In the meantime, public health officials should also consider devoting limited resources to strategies with proven mortality benefits in both high and low income countries, such as control of risk factors like hypertension, diabetes, and tobacco use [33–37].

Similarly, we secondarily noted no overall nationwide association between income and ACS mortality. This observation stands in contrast to data from other global comparison studies that have found that cardiovascular mortality tended to be lower in higher income settings [38–40]. The lack of such an association in Brazil suggests that other factors may be more important predictors of ACS mortality in the country, or that the relationship between income and ACS-related mortality may be confounded by other variables not considered in this analysis, such as education, race, lifestyle risk factors, and medical comorbidities. Further research on the interaction between ACS mortality, income, and such confounders in Brazil are needed.

### Limitations

Due to the retrospective, observational nature of this study, the findings presented here are accompanied by some limitations. First, our findings demonstrate associations and causality cannot be determined. Thus, any locally observed association between access to testing and increased or decreased ACS mortality does not imply that the presence of such testing caused the observed local ACS mortality rate. Although DATASUS is meant to be a comprehensive and accurate database, the quality of this dataset is dependent on uniform and reliable data collection. Even if reporting of utilization of diagnostic testing were perfectly accurate, reporting of ACS-related mortality is contingent upon accurate diagnosis. It is possible, therefore, that areas with poor access to cardiac testing may be subject to lower reported rates of ACS-related mortality simply due to under-detection. Furthermore, because of the de-identified nature of the data used here, analysis of the association of an individual’s socioeconomic status with testing utilization and ACS mortality was not possible, because the sociodemographic profiles of individual patients were not available. Thus, the average income of the individual’s municipality was used as a proxy measure for socioeconomic status, but more granular data would have allowed for a more robust analysis of the interactions of personal wealth, utilization, and mortality. The use of an approach based on the 2SFCA has some limitations, particularly lack of accounting for distance decay within a catchment; and failure to allow for variable catchment sizes or variable application of distance-decay [25]. However, a key strength of the 2SFCA method is that it can be readily applied to both metropolitan and rural populations [25]. This is crucial in the Brazilian context, where almost 60% of the country territory is covered by the rain forest or rural areas. Given the fact that the application of any distance-decay function results in overcorrection in metropolitan-fringe areas when the scope of the analysis considers large segments of rural areas, we elected not to include distance-decay functions in our analyses because we felt that the 2SFCA method is the most appropriate way to model spatial accessibility across vastly different population densities and dispersions [25]. Another limitation of this study was the use of a uniform 120 km radius in calculating accessibility indices. Given that transportation infrastructure is highly variable across the country, the time required for a typical patient to travel 120 km is likely to vary considerably across municipalities. Finally, this study was limited by a lack of individual-level outcomes and income data. Since individual-level income data were not available, average income across each municipality was used in all analyses, which may have masked more complex interactions between income and healthcare access at the individual or neighborhood level. However, as Brazil has over 5500 municipalities, we felt aggregation at the municipality level was justifiable. Similarly, data regarding the mortality rates, subsequent health expenditures, and complications suffered by the individuals who underwent catheterization or stress testing are unavailable in this dataset. Such data would have allowed for an analysis of cost-effectiveness and resulted in more rigorously supported recommendations for cost-effective interventions to reduce morbidity and mortality associated with ACS in Brazil.

### Conclusion

In Brazil, great variation exists in the distribution of ACS-related mortality and the utilization of cardiac diagnostic testing. As Brazil faces rising rates of risk factors for coronary artery disease that accompany rapid development, optimizing use of resources for cardiovascular care will become only more important. Access to testing was limited largely to specific developed areas, but increased access to such testing was overall not associated with geographically consistent reduction in ACS mortality. Given the lack of mortality benefit observed in this study, consistent with the results of analyses in high income countries, any expansion of advanced cardiac diagnostic testing in Brazil should be accompanied by rigorous post-expansion analysis to measure impact on local morbidity and mortality. Future research should focus on developing evidence-based strategies for the use of advanced cardiac testing in low and middle income countries.

## Supporting information

S1 Fig T-value maps of geographic weighted regression of income and accessibility indices of cardiac diagnostic testing as predictors of ACS mortality in Brazil, 2008–2014. (TIF)
