# Supplementary figures and images for: The distribution of cardiac diagnostic testing for acute coronary syndrome in the Brazilian healthcare system: A national geospatial evaluation of health access
Source: PLoS One. 2019 Jan 10;14(1):e0210502. doi: 10.1371/journal.pone.0210502 (PMC6328143; doi:10.1371/journal.pone.0210502)

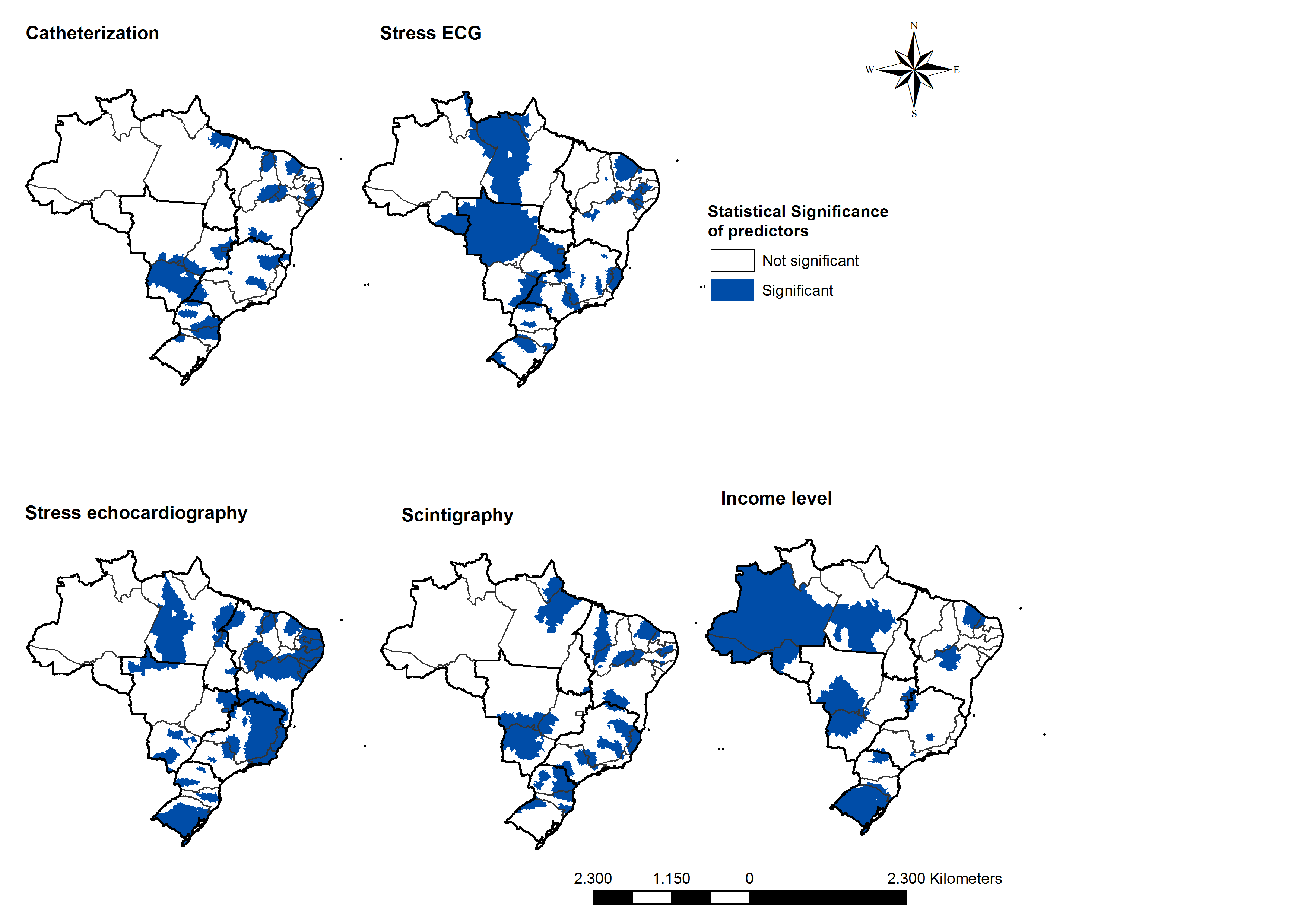

Supplement: S1 Fig — (TIF) [file pone.0210502.s001.tif]
